# Comparison of LPS and MS-induced depressive mouse model: behavior, inflammation and biochemical changes

**DOI:** 10.1186/s12888-022-04233-2

**Published:** 2022-09-05

**Authors:** Xiaojin Yu, Hui Yao, Xiaohui Zhang, Lulu Liu, Shuangmei Liu, Youjing Dong

**Affiliations:** 1 Department of Anesthesiology, Shengjing Hospital of China Medical University, No. 36 Sanhao Street, Shenyang, Liaoning 110004 P. R. China; 2 Department of Forensic Pathology, School of Forensic Medicine, China Medical University, Shenyang, Liaoning 110012 P. R. China

**Keywords:** Maternal separation, LPS, Inflammation, 5-HT, HPA axis

## Abstract

Depression is a mental disease involving complex pathophysiological mechanisms, and there are many ways to establish depressive mouse models. The purpose of this study is to comprehensively compare the behavioral changes and its mechanism induced by two different models. This study established two depressive mouse models by maternal separation (MS) or lipopolysaccharide (LPS) administration, and added fluoxetine treatment group respectively for comparison. MS induced more apparent anxiety-like behavior while LPS induced more apparent depressive-like behavior. LPS increased peripheral inflammatory factors more apparent, which were mitigated by fluoxetine. MS inhibited the 5-HT system more obviously and was relieved by fluoxetine. LPS triggered stronger immune response in the hippocampus and prefrontal cortex (PFC). MS significantly reduced the expression of neurotrophic proteins and was alleviated by fluoxetine. Overall, LPS induced stronger system inflammation, while MS impaired the function of HPA axis and 5-HT system. Our results will contribute to a deeper understanding of the pathophysiology of different stress-induced depression and will also help researchers select appropriate models of depression for their own needs.

**Supplementary Information:**

The online version contains supplementary material available at 10.1186/s12888-022-04233-2.

## Background

Major depressive disorder (MDD) is a common mental disorder characterized by depressed mood, despair and anhedonia [1]. It is estimated that 5% of adults suffer from depression disorder all over the world [2]. As a complex mental disease, the pathophysiological causes of depression remain unclear. The onset of depression involves the changes in multiple organs or systems. Etiological hypotheses such as changes in HPA axis activity [3], neurogenesis and plasticity [4], aggravated neuroinflammation [5], abnormal DNA methylation [6] have been proposed. Unfortunately, none of these theories can fully explain the pathophysiological process of depression. Risk factors contributing to depression include a family history of depression, abuse or neglect in early life, recent life stressors, and medical illness, especially those related to metabolic and autoimmune disorders [5]. Therefore, the selection of appropriate depressive model to simulate the risk factors and pathophysiological process of clinical patients is of great significance for the mechanism research of depression and the development of treatment drugs.

Early exposure to stress, such as deprivation, neglect and abuse [7, 8], have long-term effects, not only on brain function, but also on emotional development, and increase the risk of developing stress-related psychopathology in late adulthood [9, 10]. MS-induced depressive model was designed to mimic early human experiences of childhood neglect or abuse. The MS mouse model, which is widely popular, involves the pups being separated from their mother for a period of time every day after birth until the mice are weaned. Mice suffered MS tended to show cortical damage [11] or abnormal HPA axis function [12].

Numerous studies have shown a significant association between neuroinflammation and depression. Elevated levels of proinflammatory cytokines, including IL-1β, IL-6 and TNF-α, have been observed in MDD patients [13–15]. Elevated proinflammatory cytokines were also detected in brain samples from MDD patients who had committed suicide [16]. In addition, psychoneuroimmunology suggests that neuroinflammation could lead to depressive syndrome [17]. LPS, the major component of the outer membrane of Gram-negative bacteria, is widely used to induce depressive-like behavior in rodents, mimicking depressive symptoms in humans in acute infectious illness. LPS was reported to trigger systemic immune activation and immune response in brain, including production of proinflammatory cytokines [18], activation of microglia [19] and accumulation of reactive oxygen species (ROS) [20].

Given the differences in molecular mechanisms involved in different depressive models, they may also respond differently to drug therapy. Fluoxetine, a selective serotonin-reuptake inhibitor, is a traditional antidepressant [21]. In this study, fluoxetine treatment was introduced as a control to explore the responses of different depressive models to antidepressants. Multiple behavior tests were used to comprehensively compare the differences of the two models on motor capacity and emotion of mice. The peripheral and central inflammatory response, status of HPA axis, function of 5-HT system and expression of neurotrophic proteins were compared by Western blot, ELISA and immunofluorescence.

## Materials and methods

### Animals

**Fig. 1:**
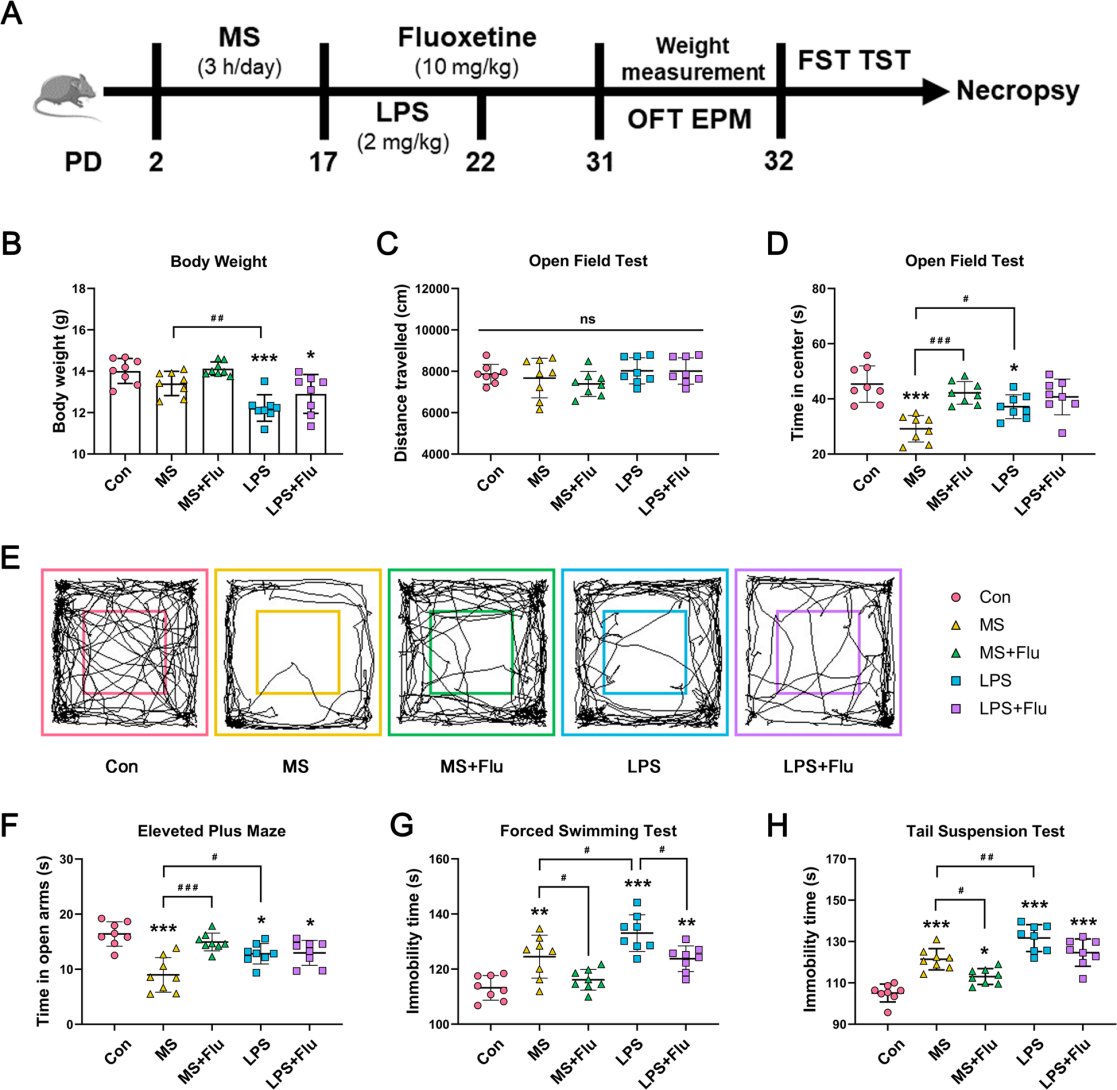
Experimental design and behavioral changes of mice after MS/LPS and fluoxetine administration. **A** Experimental design. **B** Body weight of mice on PD31. **C** Distance traveled in the OFT. **D** Time in the central area of the OFT. **E** Representative tracks of mice in the OFT. **F** Time spent in the open arms of the EPM. **G** Immobility time in the FST. **H** Immobility time in the TST. *, compared with the Con group; ^#^, compared between the groups. * / ^#^, *P* < 0.05; ** / ^##^, *P* < 0.01; *** / ^###^, *P* < 0.001. (*n* = 8 per group)

Female C57BL/6 mice (12 weeks old, weighing 21–25 g) were purchased from the Laboratory Animal Center of China Medical University. Mice were housed with free access to water and food. The indoor temperature was controlled at 21 ± 1 °C, the relative humidity was 50% ± 10%, and the light cycle was 12 hours (8,00–20:00). All animal procedures were approved by the Animal Ethics Committee of Shengjing Hospital of China Medical University. All experimental procedures were performed according to the National Institutes of Health Guide for the Care and Use of Laboratory Animals (NIH Publications No. 8023, revised 1978) and the ARRIVE guidelines on the Care and Use of Experimental Animals. Fifteen pregnant female mice were individually housed and observed daily for parturition, deemed as postnatal day (PD) 0. The entire cage was treated as one, and the 15 cages were randomly divided into a control (Con) group, a maternal separation (MS) group, a maternal separation + fluoxetine (MS + Flu) group, an LPS-treated (LPS) group and an LPS-treated + fluoxetine (LPS + Flu) group. Eight male pups from each group were randomly selected as experimental subjects on PD17. The body weight of mice was measured on PD31 before the behavioral tests. The experimental design and drug treatment schedule are shown in Fig. 1A.

### Maternal separation (MS)

Pups in MS group and MS + Flu group received maternal separation between PD2 and PD17. The method is improved according to the previous description [22]. Pups were separated from their mothers twice for 3 hours every day, during which time the mice should be observed and kept warm. Each cage of mice was removed in two batches in rotation to reduce the enhancement of maternal care. When the mice were transferred, some of the bedding with the mother’s scent was also transferred.

### LPS and fluoxetine administration

Mice in LPS group and LPS + Flu group were treated with LPS (2 mg/kg, L2880, Sigma–Aldrich, St. Louis, MO, USA) by i.p. injection for 5 consecutive days. Mice in MS + Flu group and LPS + Flu group were treated with fluoxetine hydrochloride (10 mg/kg, 343,290, Sigma–Aldrich) by i.p. injection for 14 consecutive days. Both LPS and fluoxetine were dissolved in normal saline. Mice in the Con group and MS group were treated with saline by i.p. injection during PD17 to PD31, and that in the LPS group were treated with saline by i.p. injection during PD22 to PD31.

### Behavior tests

Behavior tests were performed after drug administration and two tests were conducted daily in the following order: open field test, elevated plus-maze, forced swimming test and tail suspension test. Mice were acclimated to the testing room for 2 h before testing. Behavior test data were recorded by the SMART™ tracking software program (San Diego Instruments, San Diego, CA, USA).

#### Open field test (OFT)

The OFT was used to evaluate the depressive-like behaviors of mice performed as previously reported [23]. The OFT consisted of an empty square arena (40 × 40 × 30 cm) constructed of plastic with a white base. The central region is 20 × 20 cm. Mice were placed individually in the corner of the OFT apparatus, and spontaneous activities were recorded for 10 min using the SMART™ tracking program. After each test, the arena was cleaned with 75% ethanol to eliminate odor cues.

#### Elevated plus-maze (EPM)

The EPM test was carried out as previously reported to evaluate the anxiety-like behaviors of mice [24]. Mice were tested in a cross-shaped maze consisting of two open arms (50 × 10 cm), two closed arms (50 × 10 cm) and a central region (10 × 10 cm). Each mouse was placed in the central region of the maze and allowed to explore for 5 min. The time spent in each arm was recorded using the SMART™ tracking program. After each test, the arena was cleaned with 75% ethanol to eliminate odor cues.

#### Forced swimming test (FST)

The FST was performed as previously reported [25]. The test device consisted of a transparent cylindrical glass container (10 cm in diameter, depth of 22 cm) filled with water (23 °C to 25 °C) and a video camera in front of the container. The mice could not touch the bottom of the container with their hind legs. The test was conducted for 6 min: the first 2 min was an adaptation phase, after which the immobility of the mouse in the water was recorded for 4 min (immobility refers to the mouse’s body floating with the absence of any movement except for those necessary for keeping the nose above water). FST data were recorded by the SMART™ tracking software program.

#### Tail suspension test (TST)

The TST test was performed as previously described to assess depressive-like behavior [26]. Mice were suspended by their tail (50 cm distance from the floor) using adhesive tape at 1 cm from the tip of the tail. The TST test was conducted for 6 min, and the duration of immobility in the last 4 min was recorded. TST data were recorded by the SMART™ tracking software program.

### Animal tissue extraction

After completing the behavior tests, mice were anesthetized with isoflurane, and blood from portal vein and vena cava was centrifuged as previously reported [27] and serum samples were stored in a − 80 °C freezer. Mice were then decapitated after cervical dislocation. The right hippocampus was separated and stored in a − 80 °C freezer.

### Protein extraction and quantification

As previously reported [28], the extracted mouse tissue were detergent-extracted on ice using radioimmunoprecipitation assay (RIPA) lysis buffer (P0013B, Beyotime, Shanghai, P R China) with 1 mM phenylmethanesulfonyl fluoride (ST506, PMSF, Beyotime), disrupted on ice for 30 min, and then fragmented with ultrasonication. The lysates were collected and centrifuged at 21000 *g* for 15 min. Total proteins were quantified using a BCA protein assay kit (P0012, Beyotime).

### Elisa

ELISA was performed as previously reported [28]. The levels of IL-1β, IL-6, TNF-α, 5-HT, ACTH and CORT were investigated using assay kits (Tab S2) and following the manufacturer. Each sample (5× dilution) was used 50 μl for detection, and the absorbance at 450 nm was measured. The concentration was calculated according to the standard curve (Fig. S1-S6).

### Western blotting

Western blotting was performed as previously reported [25]. Equal amounts of protein (up to 30 μg) from the treated mice were separated by 10% SDS–PAGE and transferred to PVDF membranes. Transferred blots were blocked with nonfat milk for 2 hours and then incubated overnight at 4 °C with primary antibody (1:1000). Blots were subsequently washed and incubated with goat anti-rabbit (ZB-2301, Zsgb-Bio, 1:5000) and goat anti-mouse (ZB-2305, Zsgb-Bio, 1:5000) secondary antibodies for 2 hours. The antibodies were listed in Table S1. Protein bands were detected with ECL reagent (WBKLS0500, Merck Millipore). Chemiluminescent signals were detected and analyzed using a Tanon-5500 chemiluminescent imaging system (Tanon Science and Technology Co., Ltd., Shanghai, P R China). The intensity of the bands was analyzed using ImageJ 1.49 software (National Institutes of Health, Bethesda, MD, USA). Full-length blots/gels are presented in Supplementary Fig. S7.

### Immunofluorescence

Thirty μm thick brain tissue sections were washed with PBS. Then the sections were blocked by 8% BSA for 2 h and then treated overnight at 4 °C with primary antibody (Iba1, 1:200, Wako, Osaka, Japan). Brain sections were subsequently washed and incubated with Alexa Flour 488 (1:500, Thermo Fisher, Waltham, MA, USA) for 2 h. Sections were washed and incubated with DAPI (Absin, Shanghai, P R China) for 5 min. After washing, the sections were transferred to slides, and glass coverslips were mounted using mounting medium. Images were captured using Leica TCS SP8 laser scanning confocal microscope. The number of microglia was determined by partition counting, and the cell size was determined by Sholl analysis.

### Sholl analysis

Sholl analysis was performed as previously reported [29]. Projected z-stack image with orthogonal views were obtained by Leica TCS SP8 laser scanning confocal microscope in 1 μm steps. The z-stack images were split into single channels using Fiji and stored as 8-bit images. The estimated geometric centre was marked using the point tool in Fiji and the image was analysed with the Fiji plugins Bitmap Sholl Analysis (http://fiji.sc/Sholl_Analysis, version 3.6.8). The manual method was carried out by digitally tracing rings centred at the soma centre and intersections counted. All Sholl analyses were carried out at 2 μm intervals to a maximum radius of 24 μm.

### Statistical analysis

All data are expressed as the mean ± standard deviation (SD). Statistical analysis of data was performed using one-way analysis of variance (ANOVA) and Tukey′s multiple comparisons test. A *P value* of < 0.05 was considered significant. GraphPad Prism 8 (GraphPad Software, San Diego, CA, USA) was used for statistical analysis. All detailed statistical data are provided in Table S3.

## Results

### MS induced more apparent anxiety-like behavior while LPS induced more apparent depressive-like behavior

After 5 days of LPS injection, the mice showed significant body weight loss, while there was no statistical difference between the MS and the Con group (Fig. 1B). There was no significant difference in the total distance traveled by each group in the OFT (Fig. 1C), indicating that the behavioral differences were not caused by impaired motor ability. Compared with the Con group, mice in the MS and LPS group showed less preference in the central region of the OFT (Fig. 1D, E) and the open arms of the EPM (Fig. 1F), as well as the increased immobility time in the FST (Fig. 1G) and the TST (Fig. 1H). When compared with the MS group, mice in LPS group spent more time in the central region of the OFT (Fig. 1D, E) and the open arms of the EPM (Fig. 1F), and more immobility time in the FST (Fig. 1G) and the TST (Fig. 1H). These results suggested that both MS and LPS treatment could lead to anxiety-like and depressive-like behavior. MS induced more apparent anxiety-like behavior while LPS induced more apparent depressive-like behavior.

### The anti-anxiety and anti-depressive effects of fluoxetine were more obvious in MS-induced depressive model

Fluoxetine effectively increased the residence time of MS-treated mice in the central region of OFT (Fig. 1D) and the open arms of EPM (Fig. 1F), and reduced the immobility time in FST (Fig. 1G) and TST (Fig. 1H). When it comes to LPS-treated mice, fluoxetine only reduces the immobility time in FST (Fig. 1G). These results indicate that fluoxetine is effective for both two depressive models, but is more obvious in the MS-induced depressive model.

### LPS increased peripheral inflammatory factors more apparent, which were mitigated by fluoxetine

**Fig. 2:**
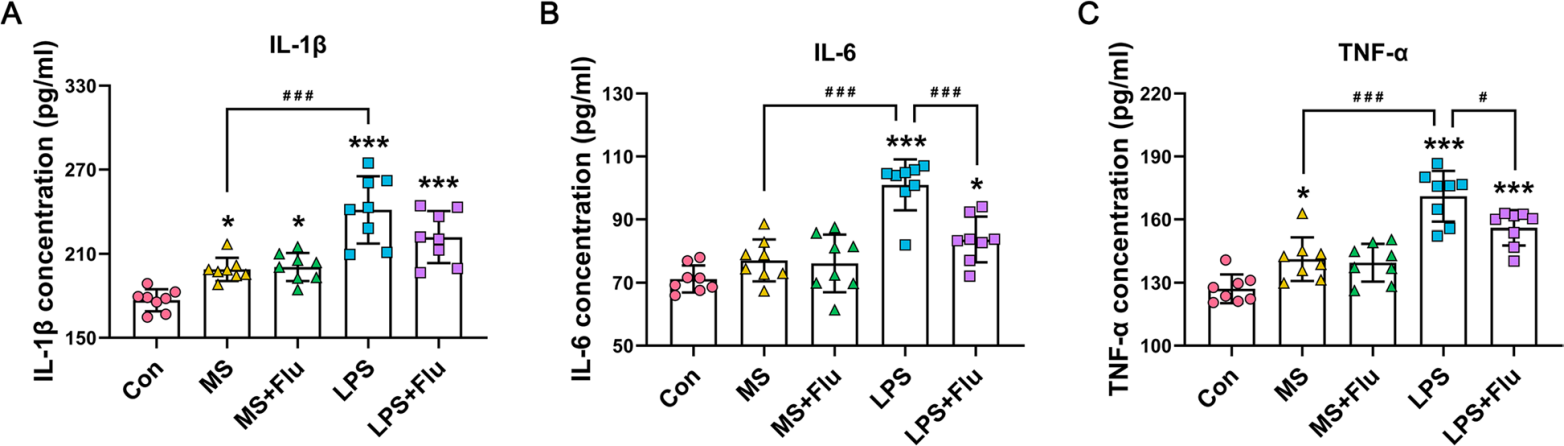
The levels of inflammatory cytokines in serum after MS/LPS and fluoxetine administration. **A** Serum IL-1β levels. **B** Serum IL-6 levels. **C** Serum TNF-α levels. *, compared with the Con group; ^#^, compared between the groups. * / ^#^, *P* < 0.05; ** / ^##^, *P* < 0.01; *** / ^###^, *P* < 0.001. (*n* = 8 per group)

Serum IL-1β (Fig. 2A) and TNF-α (Fig. 2C) of mice in the MS group were moderately increased. After LPS treatment, serum IL-1β (Fig. 2A), IL-6 (Fig. 2B) and TNF-α (Fig. 2C) were significantly increased than other groups. Fluoxetine was only weakly mitigating LPS-induced elevation of serum IL-6 (Fig. 2B) and TNF-α (Fig. 2C).

### MS activated the HPA axis which was receded by fluoxetine

**Fig. 3:**
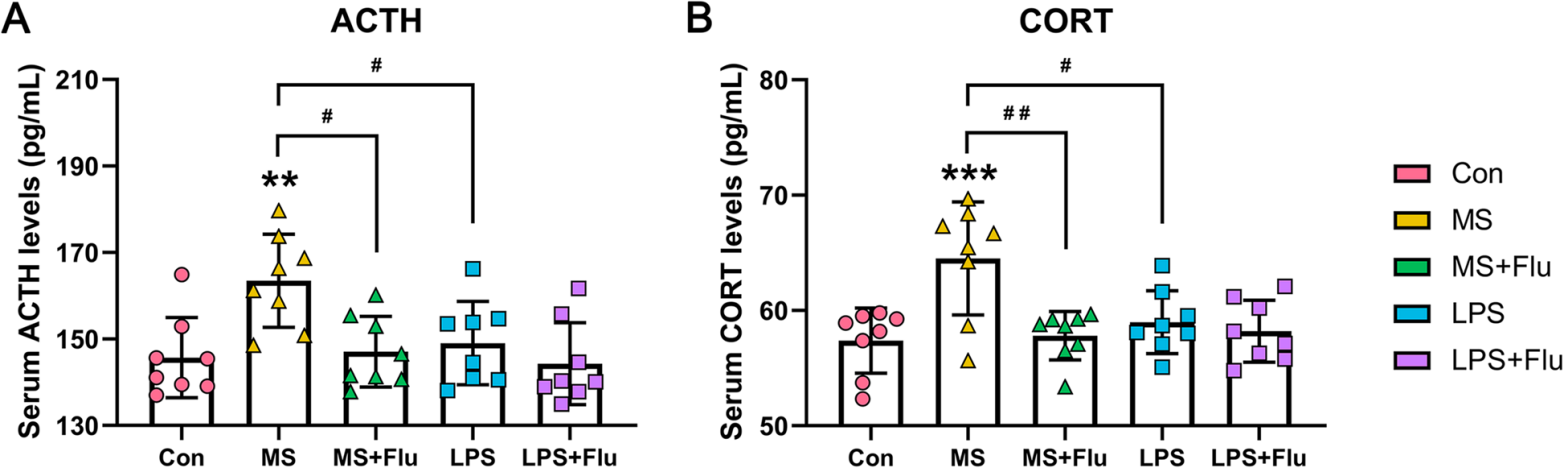
MS induced activation of HPA axis. **A** Serum ACTH levels. **B** Serum CORT levels. *, compared with the Con group; ^#^, compared between the groups. * / ^#^, *P* < 0.05; ** / ^##^, *P* < 0.01; *** / ^###^, *P* < 0.001. (*n* = 8 per group)

The activity of the HPA axis was reflected by the levels of ACTH (Fig. 3A) and CORT (Fig. 3B) in serum. There was no significant difference in serum ACTH and CORT levels between LPS group and Con group, indicating that LPS administration had no effect on the HPA axis. Serum ACTH and CORT concentration in MS group were significantly higher than that in Con group and LPS group. Fluoxetine significantly alleviated the elevation of serum ACTH and CORT induced by MS, but had no effect on the other groups.

### MS inhibited the 5-HT system more obviously which was alleviated by fluoxetine

**Fig. 4:**
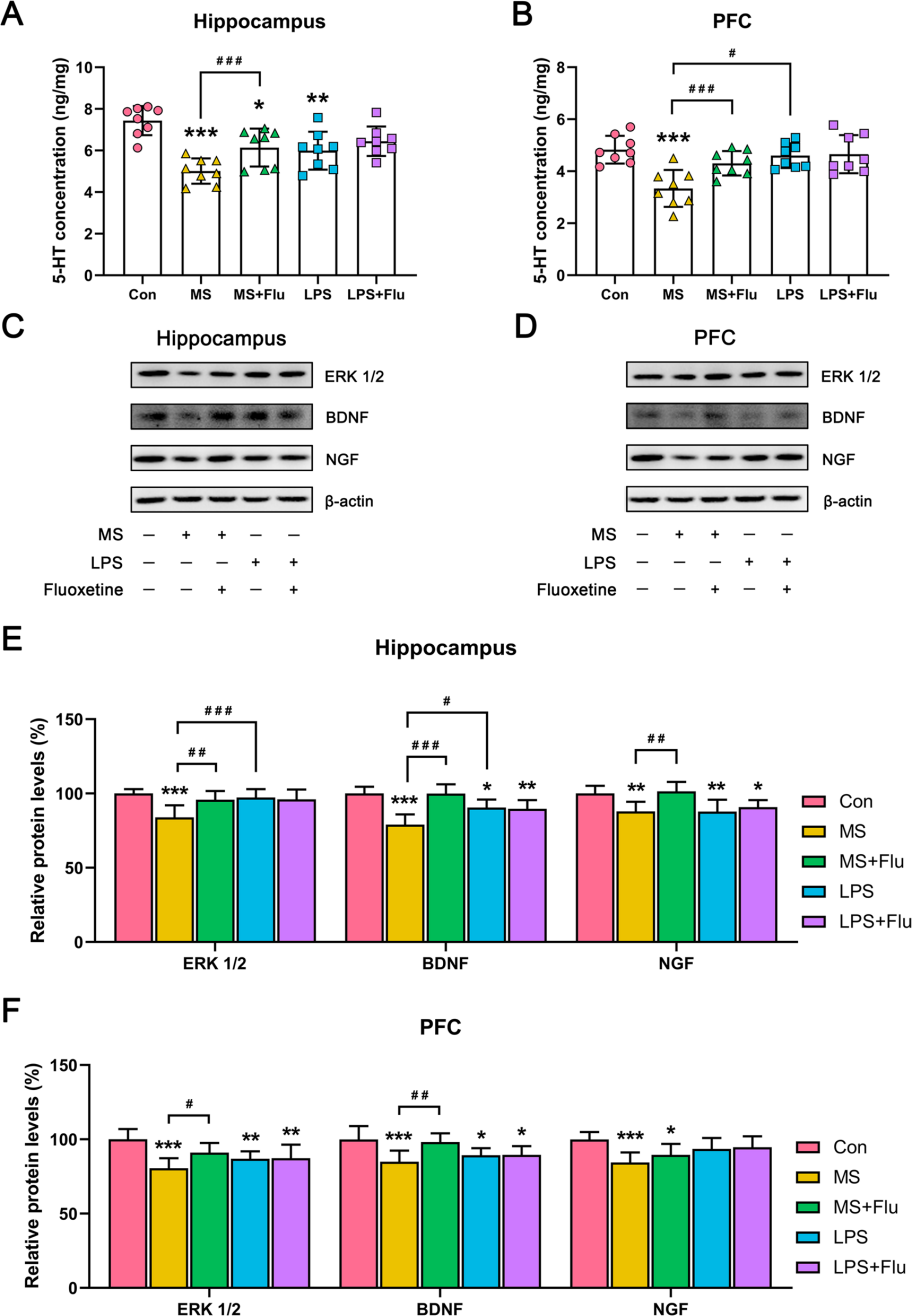
The 5-HT levels and expression of neurotrophic proteins in PFC and hippocampus. **A** 5-HT levels in hippocampus. **B** 5-HT levels in PFC. **C** Representative blot showing the expression of neurotrophic proteins in hippocampus. **D** Representative blot showing the expression of neurotrophic proteins in PFC. **E** Western blot analysis for neurotrophic proteins in hippocampus. **F** Western blot analysis for neurotrophic proteins in PFC. *, compared with the Con group; ^#^, compared between the groups. * / ^#^, *P* < 0.05; ** / ^##^, *P* < 0.01; *** / ^###^, *P* < 0.001. Full-length blots/gels are presented in Supplementary Fig. S7. (*n* = 8 per group)

In MS-induced depressive model. 5-HT levels in hippocampus (Fig. 4A) and PFC (Fig. 4B) were significantly reduced, which were effectively restored by fluoxetine (Fig. 4A, B). While in the LPS-induced depressive model, 5-HT was decreased only in the hippocampus (Fig. 4A), and fluoxetine treatment has no significant effect on this model (Fig. 4A, B).

### LPS triggered stronger immune response in the hippocampus and PFC

**Fig. 5:**
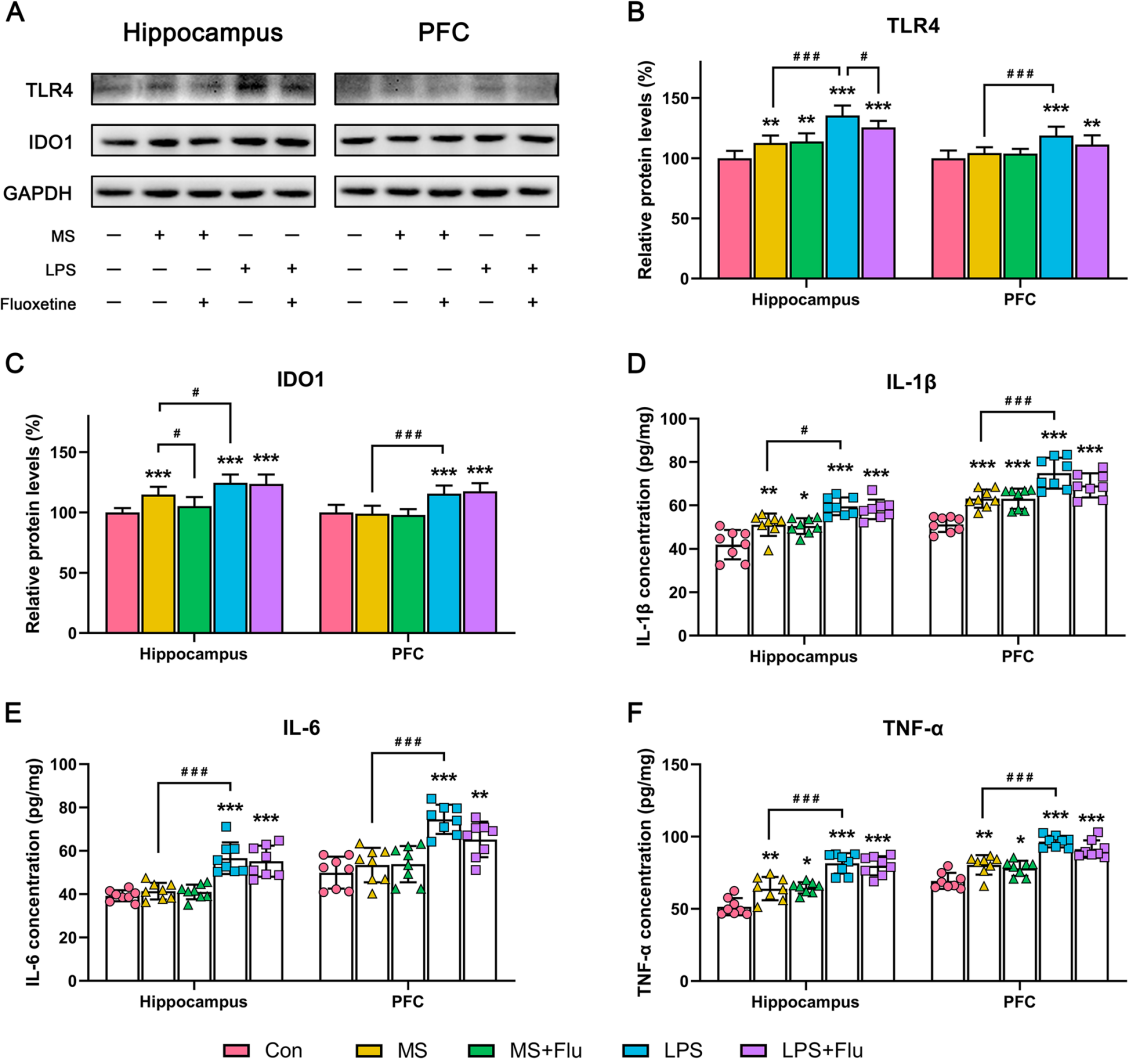
LPS triggered stronger immune response in the hippocampus and PFC. **A** Representative blot showing the expression of TLR4, IDO1 in hippocampus and PFC. **B**,**C** Western blot analysis for the expression of TLR4, IDO1 in hippocampus and PFC. **D**-**F** ELISA showing the levels of IL-1β, IL-6, TNF-αin hippocampus and PFC. Data are expressed as the mean ± SD. Statistical analysis used one-way ANOVA, *, compared with the Con group; ^#^, compared between the groups. * / ^#^, *P* < 0.05; ** / ^##^, *P* < 0.01; *** / ^###^, *P* < 0.001. Full-length blots/gels are presented in Supplementary Fig. S7. (*n* = 8 per group)

**Fig. 6:**
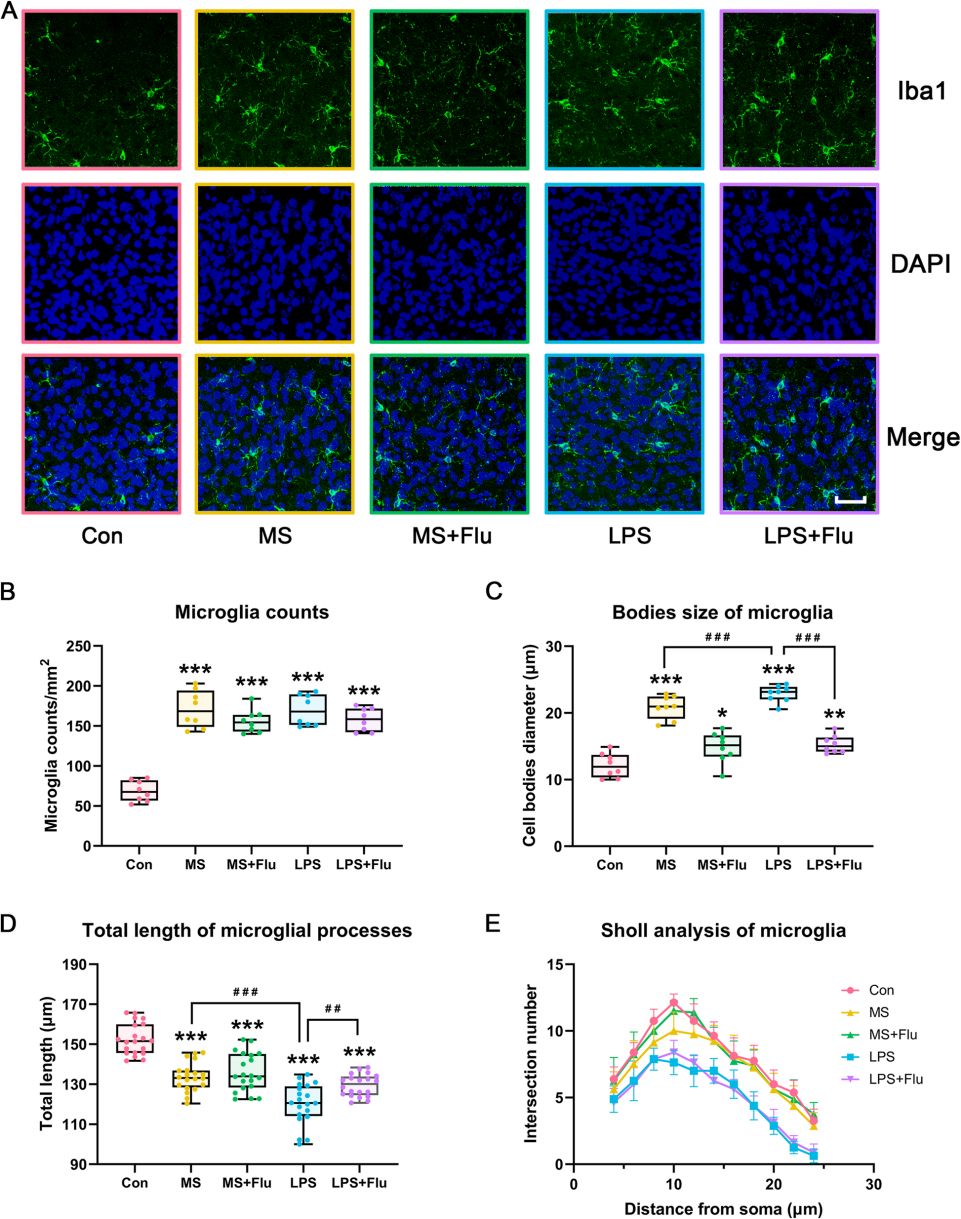
State of microglia in PFC. **A** IF showing the morphology of microglia in PFC (scale bar = 50 μm). **B** Microglia counts. **C** Bodies size of microglia. **D** Total length of microglial processes. (E) Sholl analysis of microglia. Data are expressed as the mean ± SD. Statistical analysis used one-way ANOVA, *, compared with the Con group; ^#^, compared between the groups. * / ^#^, *P* < 0.05; ** / ^##^, *P* < 0.01; *** / ^###^, *P* < 0.001. (*n* = 20 per group)

In LPS-treated mice, the expression of TLR4 (Fig. 5A, B), IDO1 (Fig. 5A, C) and levels of inflammatory cytokines IL-1β (Fig. 5D), IL-6 (Fig. 5E), TNF-α (Fig. 5F) were significantly increased, both in hippocampus and PFC. In addition, the number (Fig. 6A, B) and cell bodies size of microglia in PFC increased (Fig. 6A, C), cell complexity (Fig. 6E) and total length of microglial processes (Fig. 6D) decreased, indicating that microglia were activated in PFC. The same changes could be observed in the MS-induced depressive model, while these changes are slightly compared with the LPS-induced depressive model (Figs. 5 and 6). Fluoxetine did not significantly improve the immune response in either model, only mitigating LPS-induced TLR4 elevation (Fig. 5A, B) and MS-induced IDO1 elevation (Fig. 5A, C), in addition to a slight inhibition of microglial activation in PFC (Fig. 6).

### MS significantly reduced the expression of neurotrophic proteins and was alleviated by fluoxetine

LPS reduced the expression of BDNF and NGF in hippocampus (Fig. 4C, E), as well as the expression of ERK1/2 and BDNF in PFC (Fig. 4D, F). MS significantly reduced the expression of ERK1/2, BDNF and NGF in hippocampus (Fig. 4C, E) and PFC (Fig. 4D, F) of mice. Compared with the LPS group, MS induced a more significant decrease in ERK1/2 and BDNF in the hippocampus of mice (Fig. 4C, E). Fluoxetine only restored the reduction of ERK1/2 and BDNF in the hippocampus induced by MS (Fig. 4C, E), but had no significant effect on the reduction of neurotrophic protein in the PFC induced by MS and LPS (Fig. 4D, F).

## Discussion

In this study, MS and LPS depressive model were established to simulate two types of stress in humans, childhood neglect and inflammation stress. Behavioral tests showed that both methods were sufficient to obtain model mice with anxiety-like and depressive-like behaviors. However, when the two models were compared, it was found that there were significant differences in the degree of changes between them in the tests. These findings are consistent with many clinical or animal studies.

In this study, MS model was established based on the previously report with our modifications, because it is quite difficult to establish MS model in mice because the 3-hour separation period usually leads to an increase in maternal care [22]. In this study, we modified the MS method commonly used in rats by increasing the number of separations per day and moved offspring in two batches. The results of behavioral tests can confirm the avoiding of enhancement of maternal care and validity of the MS model.

In LPS-induced depressive model, the major changes were almost entirely in the activation of the systemic immune system. Inflammation is the result of the immune system activation. Many types of immune cells and mechanisms help maintain homeostasis, but immune disorders often lead to disease. There is increasing evidence that MDD is associated with the activation of systemic immunity. MDD patients have significantly increased proinflammation cytokines in their circulation and immune disorders in brain, also known as neuroinflammation. Inflammation cytokines are produced primarily by immune cells, including microglia in the central nervous system.

Moreover, TLR4, a ligand of LPS, activates the immune process through the NF-κB or JNK/SAPK pathways [30]. And activation of IDO1 is critical for LPS-induced microglia activation [31]. LPS-induced depressive model well mimic inflammatory stress in patients, which may account for the poor response to fluoxetine therapy. The antidepressant effect of fluoxetine is mainly through inhibiting the reuptake of 5-HT in central nervous system. Although fluoxetine has been shown to modulate immune activation, it is still controversial. In this study, fluoxetine also alleviated LPS-induced increased proinflammatory cytokine and activation of microglia, but the overall effect was not ideal. There was no significant change in TLR4 and IDO1 in PFC, so it is of reference significance to explore causes of elevated inflammatory factors. Therefore, we explored the activation state of microglia only in PFC, which is a limitation of this study.

The MS-induced depressive model was completely different, with less neuroinflammation and more mechanism involved. Childhood adversity is associated with an increased risk of depression, anxiety and substance disorders, and the molecular mechanisms behind these adverse effects are not well understood. Paraventricular nucleus of the hypothalamus (PVN) secretes corticotrophin-releasing hormone (CRH) and arginine vasopressin (AVP), which activate the anterior pituitary to induce ACTH secretion. ACTH ultimately activates the adrenal cortex to release corticosteroids, cortisol in humans and CORT in rodents [10]. The hyperactivity of HPA axis is thought to be involved in the pathogenesis of depression. In this study, increased serum ACTH and CORT indicated the hyperactivity of HPA axis. It has long been reported that stress in early life increases HPA axis activity [32, 33], and recent research demonstrated that such changes may be mediated by dysregulation of gut microbiota [34]. The changes in gut microbiota were not detected in our study, which is a limitation and will be explored in future.

5-HT, also known as serotonin, is an important neuroregulatory transmitter, and dysfunction of the 5-HT system can lead to depressive symptoms. Fluoxetine, because of its ability to inhibit 5-HT reuptake, is also used as an antidepressant. 5-HT system also regulates expression of neurotrophic proteins [35], including ERK and BDNF, and neuroplasticity, especially in early life [36]. In our study, MS significantly reduced the 5-HT levels and the expression of neurotrophic proteins in brain, and showed better improvement after fluoxetine treatment. These results suggested that an important pathophysiological changes in the MS-induced depressive model is inhibition of the 5-HT system. LPS had no significant effect on 5-HT system, but decreased the expression of neurotrophic proteins, which may be caused by neuroinflammation. MS-induced depressive model is more suitable for HPA axis and 5-HT system related research.

## Conclusions

Overall, LPS induced stronger system inflammation, while MS impaired the function of HPA axis and 5-HT system. Our results will contribute to a deeper understanding of the pathophysiology of different stress-induced depression and will also help researchers select appropriate models of depression for their own needs.

## Supplementary Information

**Additional file 1.**

## Data Availability

The datasets used and/or analyzed during the current study available from the corresponding author on reasonable request.
