# Supplementary material for: Comparison of LPS and MS-induced depressive mouse model: behavior, inflammation and biochemical changes
Source: BMC Psychiatry. 2022 Sep 5;22:590. doi: 10.1186/s12888-022-04233-2 (PMC9443001; doi:10.1186/s12888-022-04233-2)

**Supplemental information**

**Supplemental tables**

**Table. S1:** Primary antibodies used in western blot.

| ANTIBODIES | PRODUCERS | RRID |
| --- | --- | --- |
| Rabbit anti-IDO1 | Proteintech, Rosemont, IL, USA | AB_2123444 |
| Rabbit anti-BDNF | Proteintech | AB_2818984 |
| Rabbit anti-TrkB | Proteintech | AB_2155156 |
| Rabbit anti-NGF | Abcam, Cambridge, MA, USA | AB_881254 |
| Rabbit anti-TLR4 | Abcam | AB_300457 |
| Mouse anti-β-actin | Zsgb-Bio, Beijing, P R China | AB_2636897 |
| Mouse anti-GAPDH | Zsgb-Bio | AB_2747414 |

**Table. S2:** Assay kits used in ELISA.

| ANTIBODIES | PRODUCERS | IDENTIFIER |
| --- | --- | --- |
| Mouse IL-1β ELISA kit | Enzyme-linked Biotech, Shanghai, P R China | ml001814 |
| Mouse IL-6 ELISA kit | Enzyme-linked Biotech | ml002293 |
| Mouse TNF-α ELISA kit | Sangon Biotech, Shanghai, P R China | D721026 |
| Mouse 5-HT ELISA kit | Enzyme-linked Biotech | ml001891 |
| Mouse ACTH ELISA kit | Enzyme-linked Biotech | ml001895 |
| Mouse CORT ELISA kit | Enzyme-linked Biotech | ml037564 |

**Table. S3:** Statistical data.

| FIGURE | | DOF | *P* VALUE |
| --- | --- | --- | --- |
| Fig. 1B | | F _(4, 35)_ = 11.62 | < 0.001 |
| Fig. 1C | | F _(4, 35)_ = 1.225 | 0.318 |
| Fig. 1D | | F _(4, 35)_ = 10.61 | < 0.001 |
| Fig. 1F | | F _(4, 35)_ = 12.1 | < 0.001 |
| Fig. 1G | | F _(4, 35)_ = 15.25 | < 0.001 |
| Fig. 1H | | F _(4, 35)_ = 29.5 | < 0.001 |
| Fig. 2A | | F _(4, 35)_ = 21.01 | < 0.001 |
| Fig. 2B | | F _(4, 35)_ = 20.37 | < 0.001 |
| Fig. 2C | | F _(4, 35)_ = 25.27 | < 0.001 |
| Fig. 3A | | F _(4, 35)_ = 5.384 | 0.0017 |
| Fig. 3B | | F _(4, 35)_ = 6.726 | < 0.001 |
| Fig. 4A | | F _(4, 35)_ = 10.09 | < 0.001 |
| Fig. 4B | | F _(4, 35)_ = 7.969 | < 0.001 |
| Fig. 4E | ERK1/2 | F _(4, 35)_ = 8.304 | < 0.001 |
|  | BDNF | F _(4, 35)_ = 17.77 | < 0.001 |
|  | NGF | F _(4, 35)_ = 8.831 | < 0.001 |
| Fig. 4F | ERK1/2 | F _(4, 35)_ = 8.345 | < 0.001 |
|  | BDNF | F _(4, 35)_ = 7.106 | < 0.001 |
|  | NGF | F _(4, 35)_ = 5.808 | 0.0011 |
| Fig. 5B | Hippocampus | F _(4, 35)_ = 33.69 | < 0.001 |
|  | PFC | F _(4, 35)_ = 11.65 | < 0.001 |
| Fig. 5C | Hippocampus | F _(4, 35)_ = 22.04 | < 0.001 |
|  | PFC | F _(4, 35)_ = 18.96 | < 0.001 |
| Fig. 5D | Hippocampus | F _(4, 35)_ = 16.38 | < 0.001 |
|  | PFC | F _(4, 35)_ = 23.07 | < 0.001 |
| Fig. 5E | Hippocampus | F _(4, 35)_ = 20.85 | < 0.001 |
|  | PFC | F _(4, 35)_ = 13.96 | < 0.001 |
| Fig. 5F | Hippocampus | F _(4, 35)_ = 29.86 | < 0.001 |
|  | PFC | F _(4, 35)_ = 30.2 | < 0.001 |
| Fig. 7B | | F _(4, 35)_ = 41.56 | < 0.001 |

**Supplemental figures**

**Figure. S1:** Standard curve of 5-HT concentration.


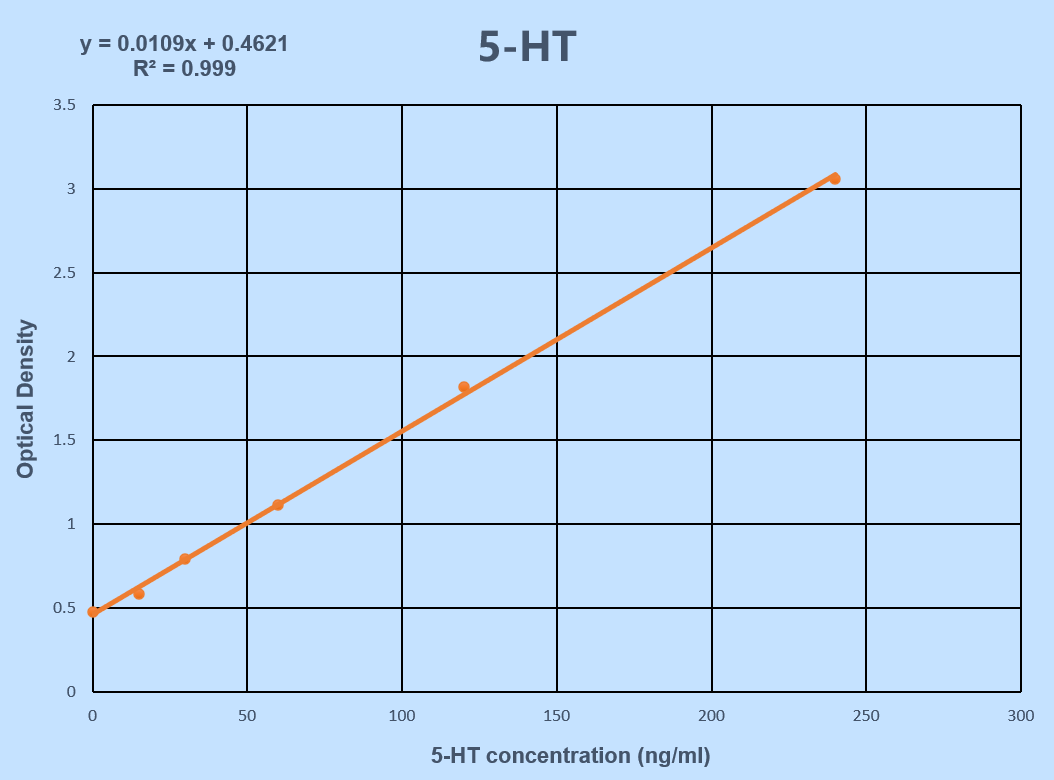


**Figure. S2:** Standard curve of IL-1β concentration.


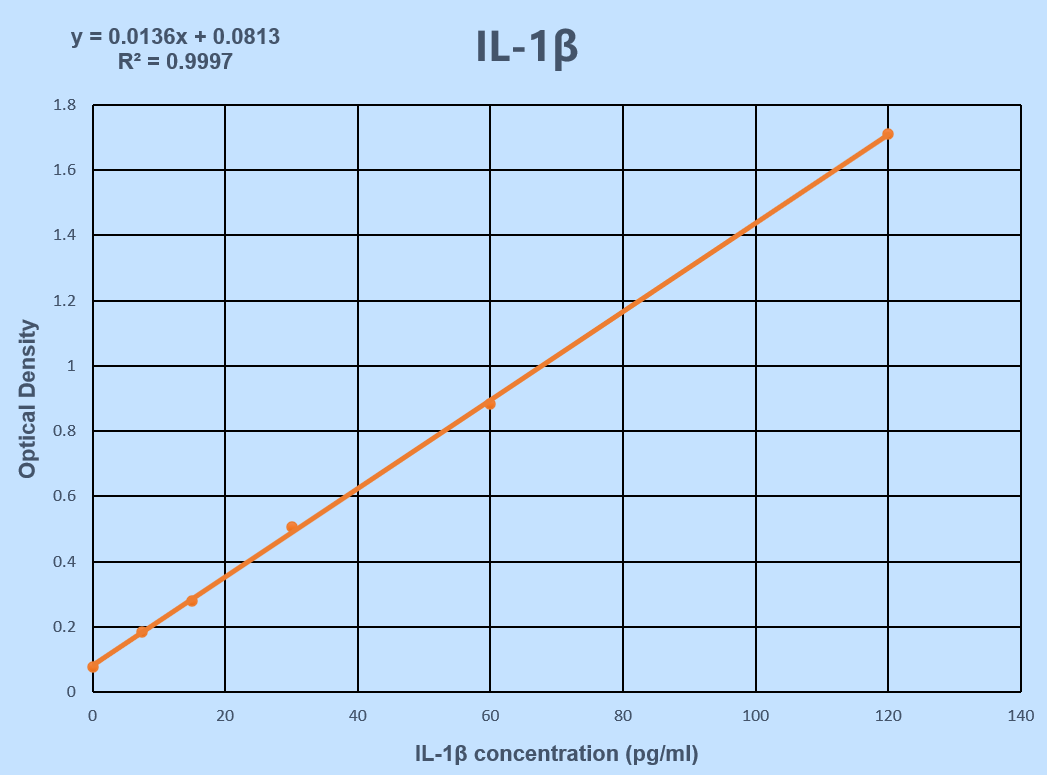


**Figure. S3:** Standard curve of IL-6 concentration.


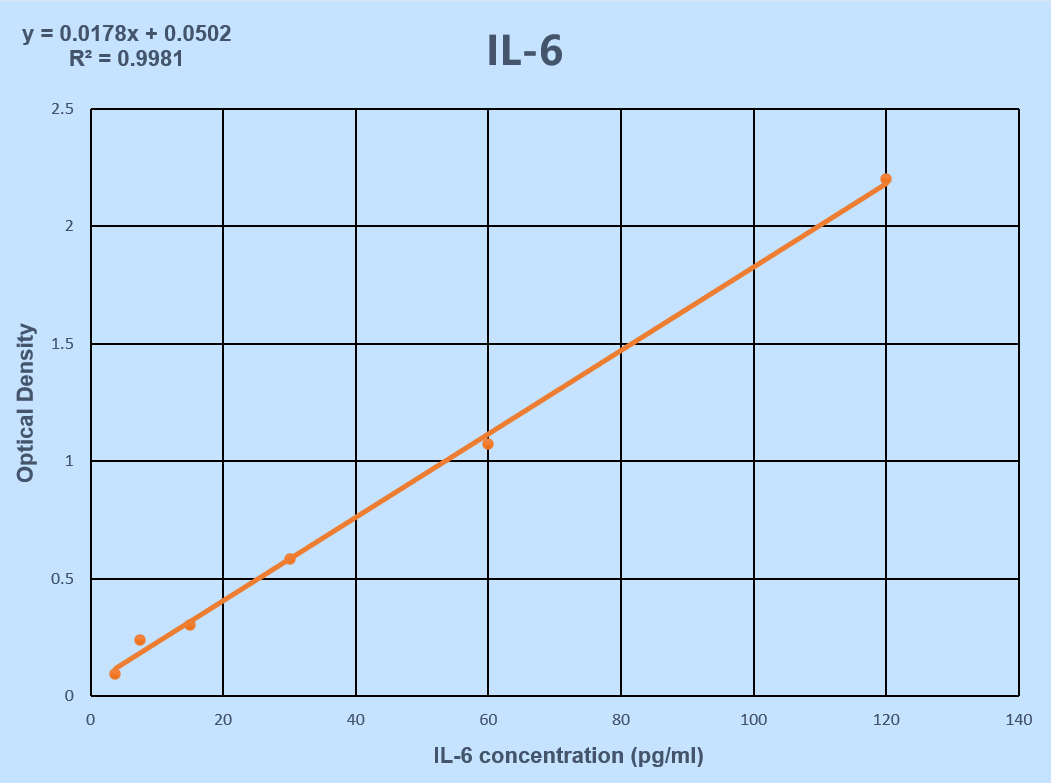


**Figure. S4:** Standard curve of TNF-α concentration.


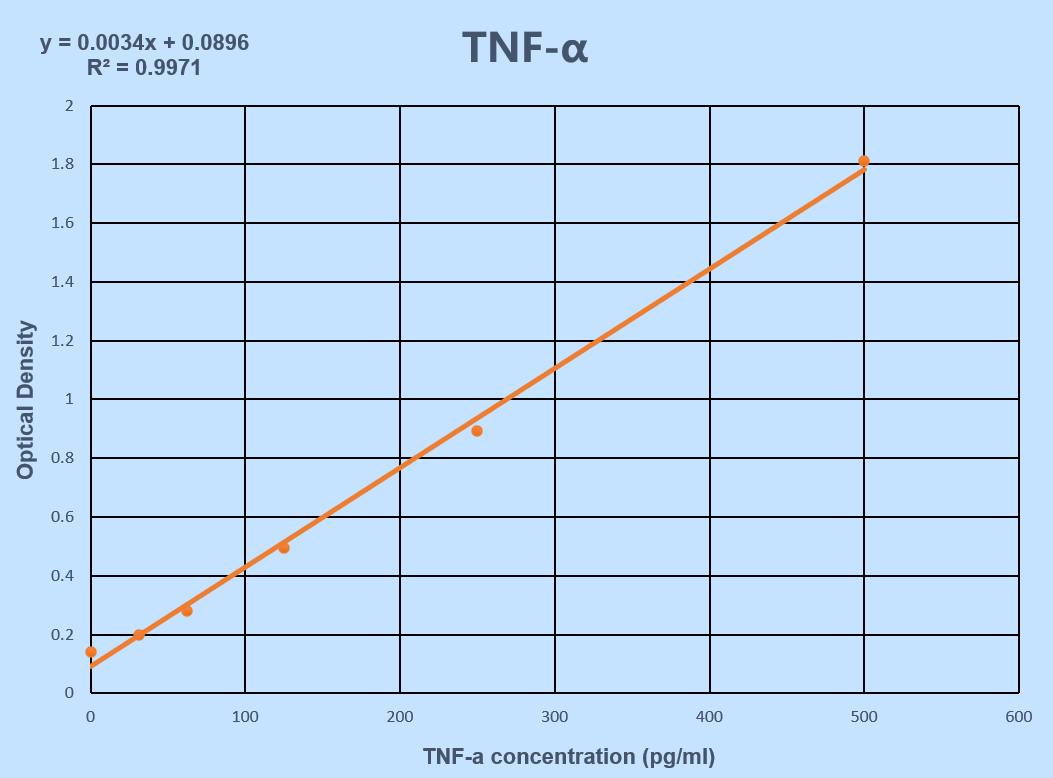


**Figure. S5:** Standard curve of ACTH concentration.

**
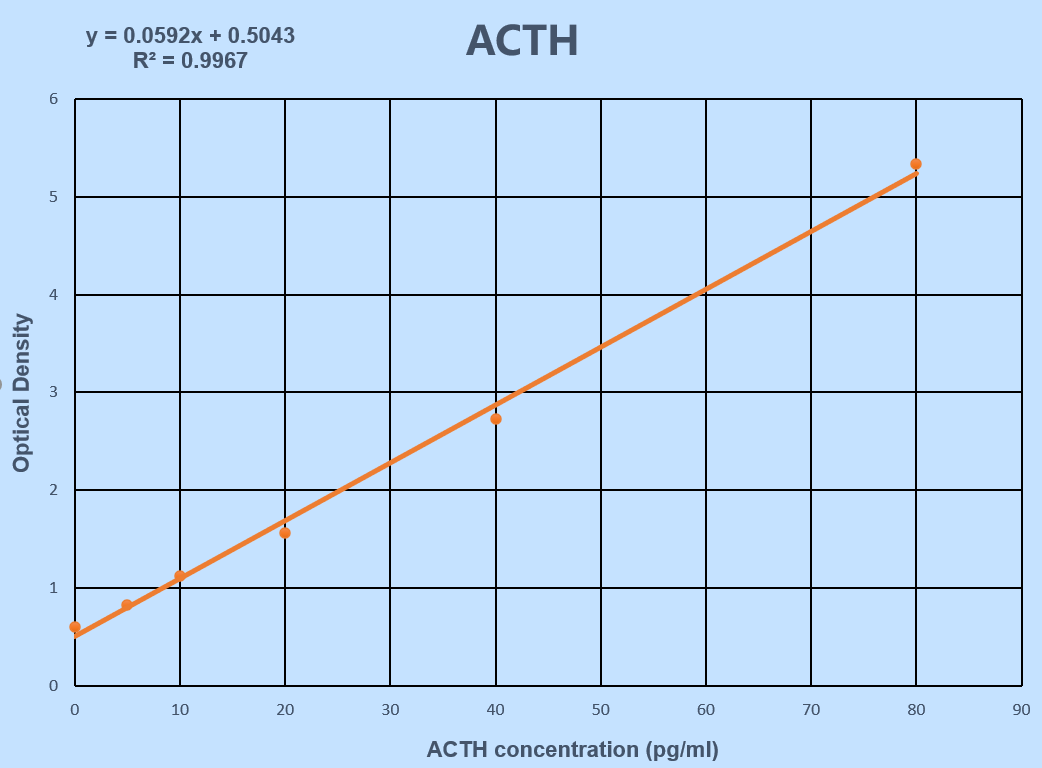
**

**Figure. S6:** Standard curve of CORT concentration.

**
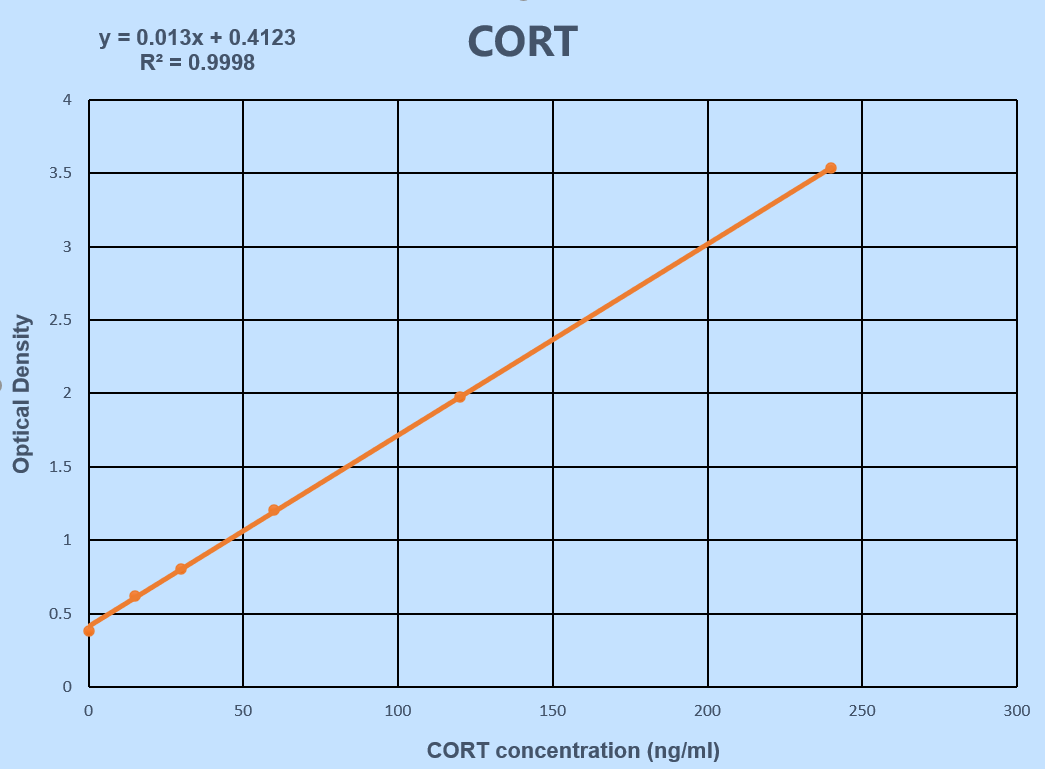
**

**Figure. S7:** Raw images of western blots from all Figures (Protein Ladder 26616 produced by ThermoFisher Scientific).


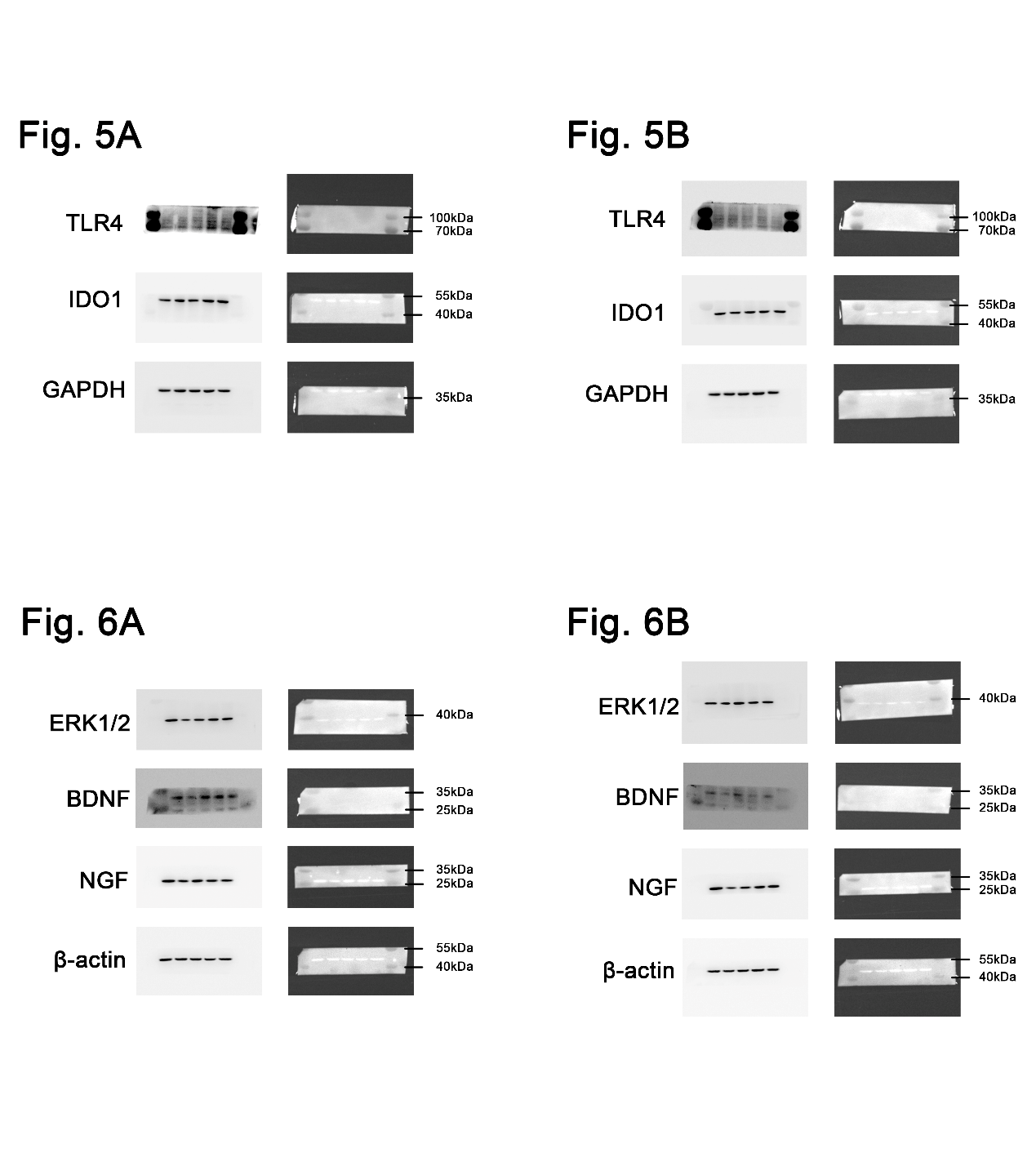

Supplement: Supplementary file 1 — Additional file 1. [file 12888_2022_4233_MOESM1_ESM.docx]
